# Cumulative Systolic Blood Pressure and Incident Stroke Type Variation by Race and Ethnicity

**DOI:** 10.1001/jamanetworkopen.2024.8502

**Published:** 2024-05-03

**Authors:** Kimson E. Johnson, Hanyu Li, Min Zhang, Mellanie V. Springer, Andrzej T. Galecki, Rachael T. Whitney, Rebecca F. Gottesman, Rodney A. Hayward, Stephen Sidney, Mitchell S. V. Elkind, W. T. Longstreth, Susan R. Heckbert, Yariv Gerber, Kevin J. Sullivan, Deborah A. Levine

**Affiliations:** 1 Department of Health Management and Policy, University of Michigan, Ann Arbor; 2 Department of Sociology, University of Michigan, Ann Arbor; 3 Department of Biostatistics, University of Michigan, Ann Arbor; 4 Vanke School of Public Health, Tsinghua University, Beijing, China; 5 Department of Neurology, University of Michigan, Ann Arbor; 6 Department of Internal Medicine and Cognitive Health Services Research Program, University of Michigan, Ann Arbor; 7 Stroke Branch, National Institute of Neurological Disorders and Stroke, Bethesda, Maryland; 8 Institute for Healthcare Policy and Innovation, University of Michigan, Ann Arbor; 9 Veterans Affairs Ann Arbor Healthcare System, Ann Arbor, Michigan; 10 Division of Research, Kaiser Permanente Northern California, Oakland; 11 Department of Neurology, Vagelos College of Physicians and Surgeons, Columbia University, New York, New York; 12 Department of Epidemiology, Mailman School of Public Health, Columbia University, New York, New York; 13 Department of Epidemiology, University of Washington, Seattle; 14 Department of Neurology, University of Washington, Seattle; 15 Department of Epidemiology and Preventive Medicine, School of Public Health, Sackler Faculty of Medicine, Tel Aviv University, Tel Aviv, Israel; 16 Lilian and Marcel Pollak Chair in Biological Anthropology, Sackler Faculty of Medicine, Tel Aviv University, Tel Aviv, Israel; 17 Department of Medicine, University of Mississippi Medical Center, Jackson; 18 Department of Neurology and Stroke Program, University of Michigan, Ann Arbor

## Abstract

**Importance:**

Stroke risk varies by systolic blood pressure (SBP), race, and ethnicity. The association between cumulative mean SBP and incident stroke type is unclear, and whether this association differs by race and ethnicity remains unknown.

**Objective:**

To examine the association between cumulative mean SBP and first incident stroke among 3 major stroke types—ischemic stroke (IS), intracerebral hemorrhage (ICH), and subarachnoid hemorrhage (SAH)—and explore how these associations vary by race and ethnicity.

**Design, Setting, and Participants:**

Individual participant data from 6 US longitudinal cohorts (January 1, 1971, to December 31, 2019) were pooled. The analysis was performed from January 1, 2022, to January 2, 2024. The median follow-up was 21.6 (IQR, 13.6-31.8) years.

**Exposure:**

Time-dependent cumulative mean SBP.

**Main Outcomes and Measures:**

The primary outcome was time from baseline visit to first incident stroke. Secondary outcomes consisted of time to first incident IS, ICH, and SAH.

**Results:**

Among 40 016 participants, 38 167 who were 18 years or older at baseline with no history of stroke and at least 1 SBP measurement before the first incident stroke were included in the analysis. Of these, 54.0% were women; 25.0% were Black, 8.9% were Hispanic of any race, and 66.2% were White. The mean (SD) age at baseline was 53.4 (17.0) years and the mean (SD) SBP at baseline was 136.9 (20.4) mm Hg. A 10–mm Hg higher cumulative mean SBP was associated with a higher risk of overall stroke (hazard ratio [HR], 1.20 [95% CI, 1.18-1.23]), IS (HR, 1.20 [95% CI, 1.17-1.22]), and ICH (HR, 1.31 [95% CI, 1.25-1.38]) but not SAH (HR, 1.13 [95% CI, 0.99-1.29]; *P* = .06). Compared with White participants, Black participants had a higher risk of IS (HR, 1.20 [95% CI, 1.09-1.33]) and ICH (HR, 1.67 [95% CI, 1.30-2.13]) and Hispanic participants of any race had a higher risk of SAH (HR, 3.81 [95% CI, 1.29-11.22]). There was no consistent evidence that race and ethnicity modified the association of cumulative mean SBP with first incident stroke and stroke type.

**Conclusions and Relevance:**

The findings of this cohort study suggest that cumulative mean SBP was associated with incident stroke type, but the associations did not differ by race and ethnicity. Culturally informed stroke prevention programs should address modifiable risk factors such as SBP along with social determinants of health and structural inequities in society.

## Key Points

**Question:**

What is the association between cumulative systolic blood pressure (SBP) and incident stroke type, and do race and ethnicity modify this association?

**Findings:**

In this cohort study using pooled data from 38 167 participants, higher cumulative SBP was associated with higher risk of overall stroke, ischemic stroke, and intracerebral hemorrhage but not subarachnoid hemorrhage. Although the risk of incident stroke type varied by race and ethnicity, little evidence suggested that race and ethnicity modified the association between cumulative SBP and incident stroke type.

**Meaning:**

These findings suggest that clinicians should emphasize SBP control as a means of stroke prevention regardless of patient race or ethnicity.

## Teaser

This pooled cohort study clarifies the association between cumulative mean systolic blood pressure and incident stroke types and explores whether race and ethnicity modify these associations.

## Introduction

High blood pressure (BP) is a major modifiable risk factor for all 3 major stroke types: ischemic stroke (IS), intracerebral hemorrhage (ICH), and subarachnoid hemorrhage (SAH). Over the past decade, guidelines for the standard of care and evaluation of BP have been updated, reclassifying hypertension guidelines.^1^ In 2017, BP guidelines were updated, redefining systolic BP (SBP) with the following measures, based on a mean of at least 2 careful readings obtained on at least 2 occasions: less than 120 mm Hg indicates normal; 120 to 129 mm Hg, elevated or prehypertension; 130 to 139 mm Hg, stage 1 hypertension; and 140 mm Hg or greater, stage 2 hypertension.^2,3^ The reclassification of SBP levels also provides additional insight for physicians and patients on differential levels of elevated cardiovascular risk.^1^ Previous research has shown that using the mean of multiple SBP measurements over time, long-term cumulative mean SBP has a greater predictive value than single SBP measurements^4,5,6^ and tends to provide better estimations of major cardiovascular events and BP control than traditional BP measures. However, the association between cumulative mean SBP and incident stroke type (IS, ICH, and SAH) is unclear.

The prevalence of hypertension and stroke is higher in Black adults than White adults in the US, likely the result of public health and health care inequities.^7^ Less clear is whether the association between cumulative SBP and incident stroke type differs by racial and ethnic group. One study^8^ found that elevated baseline SBP was associated with a higher stroke risk among Black participants than White participants; however, ischemic and hemorrhagic strokes were combined. Conversely, another study found the association between baseline hypertension (defined as a BP measurement ≥160/95 mm Hg or self-reported history of hypertension or antihypertensive drug use) and IS did not differ among Black, Caribbean Hispanic, and White participants.^9^

The objective of our study was to clarify the association between cumulative mean SBP and incident stroke types (IS, ICH, and SAH) and explore how race and ethnicity affect these associations. We hypothesized that race and ethnicity would modify the association between cumulative mean SBP and incident stroke type—specifically, that the magnitude of the association between cumulative mean SBP and stroke incidence (overall and by stroke type) would be greater in Black participants than in White participants.

## Methods

### Study Design and Participants

This pooled cohort analysis used individual participant data from 6 US prospective cohort studies with repeated measures of BP: the Atherosclerosis Risk in Communities Study (ARIC), Coronary Artery Risk Development in Young Adults Study (CARDIA), Cardiovascular Health Study (CHS), Framingham Offspring Study (FOS), Multi-Ethnic Study of Atherosclerosis (MESA), and Northern Manhattan Study (NOMAS).^5,6,10,11,12,13^ Dates covered across the 6 studies ranged from January 1, 1971, to December 31, 2019. These cohort studies were selected because they (1) have repeated measurements of SBP and its treatment over time; (2) ascertained expert-adjudicated incident stroke and stroke type; (3) used similar methods of BP measurement and stroke definitions to enable cross-cohort harmonization; (4) have high-quality data on stroke risk factors (confounders); and (5) have 1 or more of the following: racial diversity with or without ethnic diversity, age diversity (<45 and >80 years), or geographic diversity.^14,15,16^ Participants were included in the analysis if they were 18 years or older at cohort baseline, had an SBP measurement at cohort baseline, had at least 1 cohort study visit after baseline, and self-reported as Black race, Hispanic of any race, or White race. Participants with a history of prevalent stroke at cohort baseline were excluded.^5^ This study was approved by the University of Michigan Institutional Review Board. The cohort studies were approved by participating institutions, and participants provided written informed consent. This study followed the Strengthening the Reporting of Observational Studies in Epidemiology (STROBE) reporting guideline.

### Outcomes

Our primary time-to-event outcome was time from the baseline visit to the first incident stroke. Secondary outcomes included time to first incident IS, ICH, and SAH, regardless of whether the patient has experienced other stroke types before. Each cohort measured incident strokes during follow-up using similar protocols. Incident strokes were expert adjudicated using cohort data and medical records based on study protocols and published guidelines.^17,18,19,20,21,22^ Stroke events were defined as “rapidly developing clinical signs of focal, at times global, disturbance of cerebral function, lasting more than 24 hours or leading to death with no apparent cause other than that of vascular origin.”^23^ For fatal strokes, the medical history, hospital records, interviews with next of kin or proxies, and death certificate or National Death Index data were reviewed to adjudicate the cause of death. Experts further classified strokes as IS, ICH, or SAH; CHS included SAH in the ICH category, so CHS was omitted from SAH models..

### Measurement of Race and Ethnicity

Study participants self-reported their race and ethnicity in 1 of 3 groups: Black, Hispanic of any race, or White. The study incorporated the social construct of racial and ethnic categories to reflect general social definitions that are shaped by complex historical processes in the US.^24^ Including race and ethnicity in the analysis provides an opportunity to use empirical research to discuss how future interventions could address and help narrow the current inequitable gaps in stroke treatment and care among different racial and ethnic groups.^25^ By study design, ARIC, CARDIA, CHS, MESA, and NOMAS recruited Black and White participants, whereas MESA and NOMAS also recruited Hispanic participants. The CHS had a small number of self-reported Hispanic participants of any race (n = 59). Participants in the FOS were included because they provide geographic and age diversity and contribute information and precision for the estimates of time to stroke type in White participants and the estimates of stroke risk associated with SBP.

### Measurement of BP

Each cohort study measured SBP during in-person visits using similar standard protocols and equipment.^5,6,10,11,12,13^ We calculated the time-dependent cumulative mean of all SBPs (ie, a time-varying running mean) updated to reflect each SBP measurement before the event.

### Covariates

Sociodemographic covariates included age, gender, sex, educational level, and cohort study. Vascular risk factors included current cigarette smoking, physical activity, body mass index, waist circumference, history of atrial fibrillation, fasting glucose level, low-density lipoprotein cholesterol level, alcohol use, and time-dependent antihypertensive medication use. Participants with no antihypertensive medication use recorded at all visits were considered nonusers. All covariates except for antihypertensive medication use were measured at baseline.

### Statistical Analysis

#### Main Analysis

Data were analyzed from January 1, 2022, to January 2, 2024. A subgroup analysis approach using Kaplan-Meier methods and log-rank tests for overall stroke and stroke type were used to evaluate the cumulative incidence of first event and compare curves stratified by the 3 race and ethnicity groups.^26,27^ We constructed multivariable Cox proportional hazards models to test the association between time-dependent cumulative mean SBP and the time-to-event outcomes adjusting for covariates. Racial and ethnicity differences in the association between SBP and stroke or stroke type were examined by (1) including an SBP by race and ethnicity interaction term in the model, and (2) stratifying analyses of SBP by racial or ethnic stratum and reported using hazard ratios (HRs). We used multiple imputations (10 imputed datasets) to replace the missing covariates at baseline.^28,29^ Hazard ratios are shown for a 10–mm Hg increase in cumulative mean SBP. Analyses were performed using SAS, version 9.4 (SAS Institute Inc) and R, version 4.1.1 (R Project for Statistical Computing). Statistical significance for all analyses was set as 2-sided *P* < .05.

#### Sensitivity Analysis

To examine whether the observed differences in SBP-related stroke risk (HRs) by race and ethnicity were due to cohort variation, we separated the cohorts into 2 groups and repeated the analyses within the 2 groups separately. The first group included cohorts that did not recruit Hispanic participants by design (ARIC, CARDIA, CHS, and FOS, excluding the 59 Hispanic participants in CHS). The second group included cohorts that recruited Hispanic participants by study design (MESA and NOMAS). Further details are provided in the eTable in Supplement 1.

## Results

**Figure 1.**
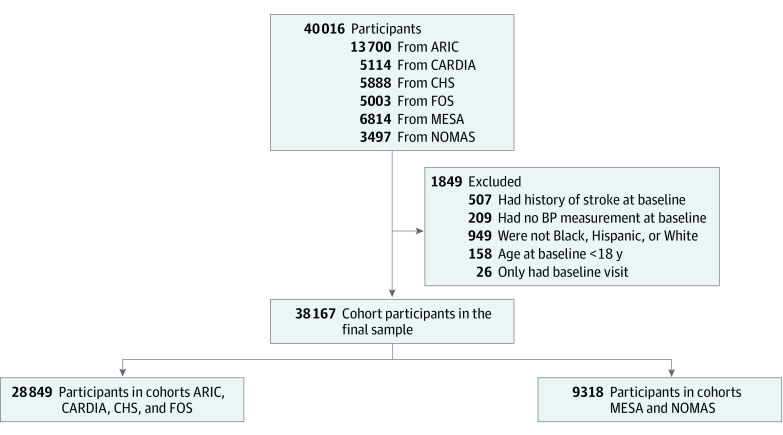
Study Flow Diagram ARIC indicates Atherosclerosis Risk in Communities Study; BP, blood pressure; CARDIA, Coronary Artery Risk Development in Young Adults Study; CHS, Cardiovascular Health Study; FOS, Framingham Offspring Study; MESA, Multi-Ethnic Study of Atherosclerosis; and NOMAS, Northern Manhattan Study. The eTable in Supplement 1 includes a sensitivity analysis restricted to the 4 cohorts that recruited Black and White participants by design (n = 28 849) and the 2 cohorts that recruited Black, Hispanic, and White participants by design (n = 9318).

The study sample included 38 167 participants consisting of 20 819 women (54.5%) and 17 348 men (45.5%). In terms of race and ethnicity, 9535 participants (25.0%) identified as Black; 3380 (8.9%), Hispanic of any race; and 25 252 (66.2%), White. The median baseline SBP measurements for the 3 race and ethnicity groups were 137.7 (IQR, 127.3-150.7) mm Hg for Black participants, 135.0 (IQR, 120.0-150.0) mm Hg for Hispanic participants of any race, and 134.0 (IQR, 122.0-147.0) mm Hg for White participants. Figure 1 shows the derivation of the pooled cohort. The Table presents the participant characteristics and the counts for the first overall stroke by racial and ethnic groups. The mean (SD) baseline age was 53.4 (17.0) years and the mean (SD) SBP at baseline was 136.9 (20.4) mm Hg. Over a median follow-up of 21.6 (IQR, 13.6-31.8) years, 3502 participants experienced a stroke: 847 Black, 342 Hispanic of any race, and 2313 White. Of the 3502 incident strokes, IS (2918 [83.3%]) was more common than ICH (392 [11.2%]), SAH (95 [2.7%]), and unknown or other (97 [2.8%]). A similar pattern was seen for the 3 race and ethnicity groups.

**Table.** Baseline Characteristics and Stroke Incidence Among Participants

Characteristics	Participant group^a^			
	All (N = 38 167)	Black (n = 9535)	Hispanic of any race (n = 3380)	White (n = 25 252)
Follow-up time, median (IQR), y	21.6 (13.6-31.8)	20.3 (12.9-31.1)	14.1 (10.1-18.8)	25.2 (14.2-32.3)
Follow-up time, range, y	0-47.3	0-46.5	0-26.5	0-47.3
SBP at cohort baseline, mean (SD), mm Hg^b^	136.9 (20.4)	140.5 (20.6)	136.2 (22.8)	135.6 (19.9)
First-time incident stroke types^c^				
Overall	3502 (100)	847 (100)	342 (100)	2313 (100)
IS	2918 (83.3)	690 (81.5)	285 (83.3)	1943 (84.0)
ICH	392 (11.2)	111 (13.1)	34 (9.9)	247 (10.7)
SAH	95 (2.7)	25 (3.0)	14 (4.1)	56 (2.4)
Unknown or other	97 (2.8)	21 (2.5)	9 (2.6)	67 (2.9)
Demographics				
Age at first SBP measurement, mean (SD), y	53.4 (17.0)	50.5 (18.7)	64.1 (10.1)	53.0 (16.5)
Sex				
Women	20 819 (54.5)	5621 (59.0)	1953 (57.8)	13 245 (52.5)
Men	17 348 (45.5)	3914 (41.0)	1427 (42.2)	12 007 (47.5)
Educational level at first SBP assessment				
College graduate or more	9794 (29.4)	2222 (23.4)	258 (7.6)	7314 (35.9)
Some college but no degree	6219 (18.7)	2119 (22.3)	496 (14.7)	3604 (17.7)
Completed high school	9091 (27.3)	2462 (25.9)	525 (15.5)	6104 (29.9)
Grades 9-11	4035 (12.1)	1555 (16.4)	446 (13.2)	2034 (10.0)
Grade 8 or less	4122 (12.4)	1139 (12.0)	1654 (48.9)	1329 (6.5)
Current cigarette smoking	9245 (24.3)	2539 (26.8)	467 (13.8)	6239 (24.8)
Any physical activity^d^	26 305 (79.2)	7133 (75.2)	2425 (72.1)	16 747 (82.2)
Body mass index, mean (SD)^e^	27.0 (5.3)	28.4 (6.3)	28.8 (5.1)	26.3 (4.8)
Waist circumference, mean (SD), cm	93.9 (15.2)	93.6 (17.1)	96.9 (12.7)	93.5 (14.5)
History of atrial fibrillation	367 (1.0)	77 (0.8)	53 (1.6)	237 (0.9)
Fasting glucose level, mean (SD), mg/dL	102.9 (37.8)	104.0 (47.1)	105.7 (45.5)	101.9 (31.0)
LDL cholesterol level, mean (SD), mg/dL	127.5 (37.3)	123.8 (39.1)	124.3 (34.9)	129.3 (36.9)
Alcohol use, No. drinks/wk^f^				
None	16 488 (46.0)	5091 (58.9)	1347 (50.3)	10 050 (41.0)
1-6	12 176 (34.0)	2313 (26.7)	1032 (38.6)	8831 (36.0)
7-13	3881 (10.8)	699 (8.1)	171 (6.4)	3011 (12.3)
>14	3280 (9.2)	545 (6.3)	127 (4.7)	2608 (10.6)
Antihypertensive medication use^d^	9978 (30.2)	3299 (47.4)	1344 (39.8)	5335 (23.5)

Abbreviations: ICH, intracerebral hemorrhage; IS, ischemic stroke; LDL, low-density lipoprotein; SAH, subarachnoid hemorrhage; SBP, systolic blood pressure.

SI conversion factors: To convert LDL cholesterol to mmol/L, multiply by 0.0259; glucose to mmol/L, multiply by 0.0555.

^a^ Unless otherwise indicated, data are expressed as No. (%) of participants with available data.

^b^ Calculated using the time-dependent cumulative mean of all SBP measurements (ie, a time-varying running mean) at each SBP measurement before the event.

^c^ Denominator is the total number of strokes. The Cardiovascular Health Study (CHS) cohort data included SAH in the ICH category; therefore, CHS participants are not included in the SAH models.

^d^ Includes participants with available data.

^e^ Calculated as weight in kilograms divided by height in meters squared.

^f^ One drink equals approximately 14 grams of alcohol in our harmonized variable based on a self-report of alcohol use.

### Absolute Risks for Overall Stroke and Stroke Type

**Figure 2.**
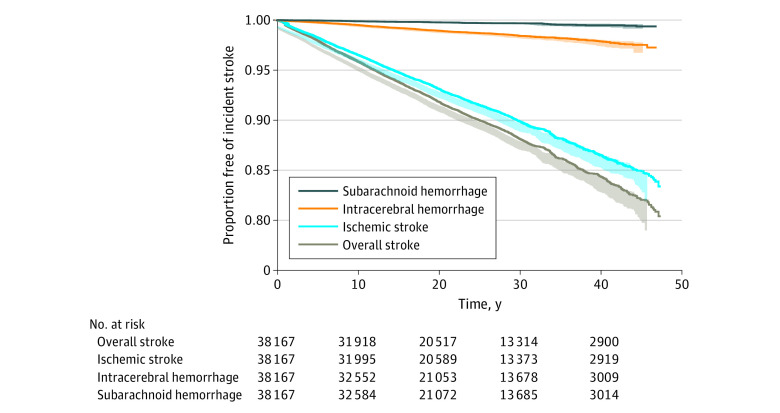
Absolute Risks for Overall Stroke and Stroke Type

Among the 3 stroke types, the proportion remaining free of incident stroke type was higher among participants who experienced SAH and ICH compared with those who experienced IS. Figure 2 presents the Kaplan-Meier survival curves for time to incident stroke type.

### Association of SBP With Overall Stroke and Stroke Type

**Figure 3.**
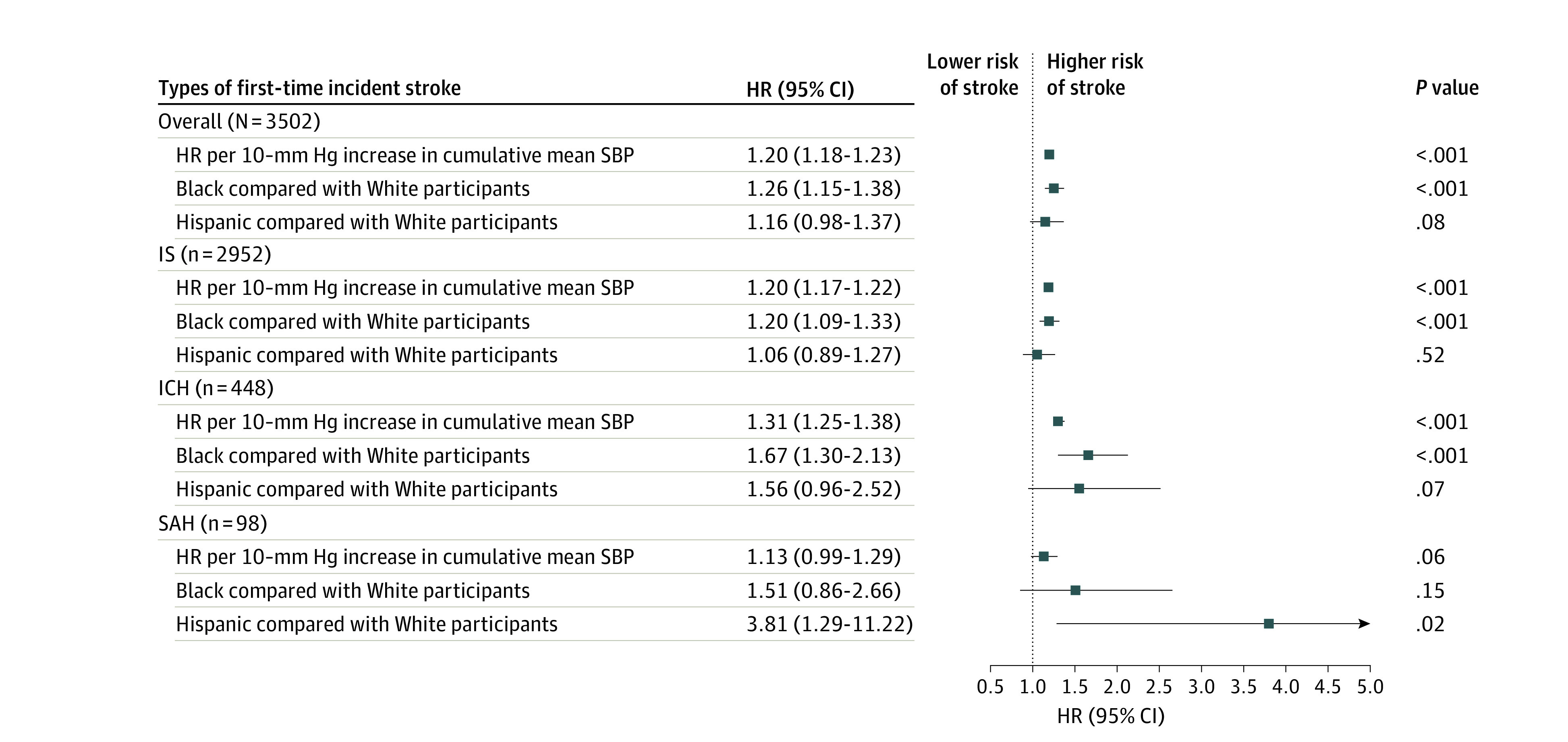
Cumulative Mean Systolic Blood Pressure and Time to Incident Stroke Models for overall stroke, ischemic stroke (IS), and intracerebral hemorrhage (ICH) used multivariable adjusted Cox proportional hazard regression. HR indicates hazard ratio; SAH, subarachnoid hemorrhage; SBP, systolic blood pressure.

Figure 3 shows the HRs of cumulative mean SBP for the incidence of overall stroke and stroke subtypes adjusted for covariates: 3502 participants had a stroke, 2952 had an IS, 448 had an ICH, and 98 had an SAH. A 10–mm Hg higher cumulative mean SBP was associated with a 20% higher risk of overall stroke (HR, 1.20 [95% CI, 1.18-1.23]), 20% higher risk of IS (HR, 1.20 [95% CI, 1.17-1.22]), and 31% higher risk of ICH (HR, 1.31 [95% CI, 1.25-1.38]). The 13% higher risk of SAH associated with a 10–mm Hg higher cumulative mean SBP was not significant (HR, 1.13 [95% CI, 0.99-1.29]; *P* = .06).

### Incidence of Stroke and Stroke Type by Race and Ethnicity

Figure 3 also shows adjusted HRs for incident stroke by race and ethnicity. After adjusting for covariates, compared with White participants, Black participants had a 20% higher risk of IS (HR, 1.20 [95% CI, 1.09-1.33]) and a 67% higher risk of ICH (HR, 1.67 [95% CI, 1.30-2.13]) but not of SAH (HR, 1.51 [95% CI, 0.86-2.66). Compared with White participants, Hispanic participants of any race had a 281% higher risk of SAH (HR, 3.81 [95% CI, 1.29-11.22]), but not other stroke types. Kaplan-Meier survival curves for time to incident stroke type by race and ethnicity representing unadjusted HRs can be found in the eFigure in Supplement 1).

### Exploring Whether Race and Ethnicity Modifies the Association Between SBP and Stroke

When all 6 cohorts were examined, race and ethnicity did not modify the association between cumulative mean SBP and risk of IS (race/ethnicity × SBP interaction term, *P* = .85) or SAH (race/ethnicity × SBP interaction term, *P* = .89) but did modify the association for ICH (race/ethnicity × SBP interaction term, *P* = .02) (eTable in Supplement 1). An analysis restricted to the 4 cohorts that recruited Black and White participants by design (n = 28 849) similarly suggested that race did not modify the association between cumulative mean SBP and risk of overall stroke (race/ethnicity × SBP interaction term, *P* = .03), IS (race/ethnicity × SBP interaction term, *P* = .47), or SAH (race/ethnicity × SBP interaction term, *P* = .82) but did modify the risk of ICH (race/ethnicity × SBP interaction term, *P* = .001) (eTable in Supplement 1). In analyses restricted to the 2 cohorts that recruited Black, Hispanic, and White participants by design (n = 9318), we found that race and ethnicity did not modify the association between cumulative mean SBP and risk of ICH (race/ethnicity × SBP interaction term, *P* = .88) or SAH (race/ethnicity × SBP interaction term, *P* = .11) (eTable in Supplement 1). Marginal evidence suggested that race and ethnicity modified the association between cumulative mean SBP and risk of overall stroke (race/ethnicity × SBP interaction term, *P* = .06) and IS (race/ethnicity × SBP interaction term, *P* = .06) (eTable in Supplement 1). Hence, these analyses do not provide consistent evidence that race and ethnicity modified the association of cumulative mean SBP with incident stroke and stroke type.

## Discussion

In this pooled analysis of 38 167 participants from 6 prospective cohort studies, a 10–mm Hg higher cumulative mean SBP was associated with a significantly higher risk of ICH at 31% and higher risk of IS at 20%. The 13% higher risk of SAH associated with a 10–mm Hg higher cumulative mean SBP was not significant, possibly reflecting the small number of SAH cases (n = 98) compared with IS cases (n = 2952) and ICH cases (n = 448). We found no consistent evidence that race and ethnicity modified the association between cumulative SBP and first incident stroke overall or stroke type.

Our results are consistent with previous studies showing that higher BP is associated with higher risk of ICH than IS.^30,31,32^ Few population-based studies examined SAH risk because SAH is relatively rare and harder to study among the stroke types. Some population studies combine ICH and SAH into a single category. Previous research^33,34^ found that higher SBP levels were associated with a higher risk of SAH. Our results might differ because we had only 98 cases of SAH, and 1 cohort (CHS) combined SAH and ICH. Another potential explanation is that SAH often occurs at younger ages than other stroke types, and most cohorts recruited participants 40 years or older or 65 years or older.^33,35^

We found that the risk of stroke type varied by race and ethnicity. Compared with White participants, Black participants had a 20% higher risk of IS and 67% higher risk of ICH but similar risk of SAH. Compared with White participants, Hispanic participants of any race had a 3-fold higher risk of SAH (HR, 3.81), but similar risk of other stroke types. Previous research has found that Black participants have higher ICH incidence than White participants of similar age, primarily due to Black participants’ greater incidence and prevalence of hypertension.^36,37,38^ Black participants have also been found to have a higher incidence of IS than White participants.^39^ A few community studies have found that Hispanic^40^ and Mexican American^41^ participants had a higher incidence of SAH than non-Hispanic White participants. Our study extends previous work^42^ by examining the association between long-term cumulative mean SBP and incident stroke types in broader age ranges across the lifespan in a diverse sample of participants that included Hispanic participants of any race.

We found no consistent evidence that race and ethnicity modified the association between cumulative mean SBP with first incident stroke and stroke type, contrary to our hypothesis. Although the analysis of the 6 cohorts suggested that the cumulative SBP-associated risk of ICH differed by race and ethnicity, the sensitivity analysis results suggest that cohort variation rather than true racial and ethnic differences contributed to differences in cumulative SBP-associated risk of ICH between racial and ethnic groups. The race × SBP interaction term for ICH was significant in the subgroup of cohorts that recruited Black and White participants by design, but the race and ethnicity × SBP interaction term for ICH was not significant in the cohorts that recruited Black participants, Hispanic participants of any race, and White participants by study design (MESA and NOMAS). The differences in the race and ethnicity × SBP interaction term for the MESA and NOMAS cohort studies could be due to cohort differences, not race and ethnicity differences. Possibly, White participants’ stroke risks in MESA and NOMAS differ from those in the other 4 cohorts. Alternatively, it is possible that the statistical power for testing interactions was much smaller in the subgroup analysis of MESA and NOMAS than in the subgroup analysis of the 4 cohorts (ARIC, CARDIA, CHS, and FOS), which featured a larger sample size.

### Strengths and Limitations

Our study has several strengths. We used data collected across the US to widen our findings’ generalizability by pooling data from 6 population cohort studies that have diversity in age, geographic diversity, and race and/or ethnicity. Two advantages of a pooled cohort analysis include the increased study power to examine effect modification and the ability to examine risks across large samples of participants with heterogeneous exposures.^43^

Our study also has potential limitations. Although we adjusted for educational level, we did not include other socioeconomic factors that could be potential confounders, such as income, because they were unavailable for all cohorts at or before the first incidence of stroke.^44,45^ Although our study examines a broad age range of participants, our study examined participants’ age at baseline only. We did not investigate the influence of age on the associations among cumulative mean SBP, race and ethnicity, and incident stroke risk. Future research should examine the effect of age on the association between cumulative mean SBP and stroke risk among diverse populations, as advanced age has been identified as a risk factor for stroke incidence. The number of cases with SAH was small, limiting the ability to detect an association with cumulative mean SBP.

## Conclusions

In this cohort study of 38 167 participants, our study results have clinical and research implications. Our results suggest that cumulative mean SBP was a potent modifiable risk factor for stroke, IS, and ICH. However, since 2007 to 2008, BP control has not improved and has worsened.^46^ Our results suggest that early diagnosis and sustained treatment of elevated BP and BP control over the life course were critical to prevent stroke, IS, and ICH, especially in Black and Hispanic patients who are more likely to have undiagnosed and uncontrolled high BP than White patients.^47,48,49^ Although self-monitoring of BP improves BP control and is cost-effective, it is an underused tool, and cost is a barrier, making patient education and greater insurance coverage priorities.^50,51^ Although we found no clear evidence that the association between SBP on incident stroke type differed by race and ethnicity, stroke risk varied by race and ethnicity, with Black participants having higher IS and ICH risk and Hispanic participants having higher ICH risk than White participants.^39,52^ Examining racial inequities advances our understanding of the social, economic, and political structures that affect health behaviors, experiences, and incident stroke for racial and ethnic minority groups.^24^ Our findings highlight the importance of providing culturally informed stroke prevention programs addressing modifiable risk factors such as BP, along with social determinants of health and structural inequities in society.

## Notes

Supplement 1. **eFigure.** Absolute Risk for Overall Stroke and Stroke Type by Race and Ethnicity **eTable.** Sensitivity Analysis of Association Between Cumulative Mean Systolic Blood Pressure and Time to Incident Stroke in Pooled Cohort Sample by Cohort Subgroup, 1971 to 2019

Supplement 2.
Data Sharing Statement
