# Supplementary material for: Cumulative Systolic Blood Pressure and Incident Stroke Type Variation by Race and Ethnicity
Source: JAMA Netw Open. 2024 May 3;7(5):e248502. doi: 10.1001/jamanetworkopen.2024.8502 (PMC11069082; doi:10.1001/jamanetworkopen.2024.8502)
Supplement: Supplement 1. — eFigure. Absolute Risk for Overall Stroke and Stroke Type by Race and Ethnicity eTable. Sensitivity Analysis of Association Between Cumulative Mean Systolic Blood Pressure and Time to Incident Stroke in Pooled Cohort Sample by Cohort Subgroup, 1971 to 2019 [file jamanetwopen-e248502-s001.pdf]

Supplementary Online Content

Johnson KE, Li H, Zhang M, et al. Cumulative systolic blood pressure and incident stroke type variation by race and ethnicity. *JAMA Netw Open*. 2024;7(4):e248502. doi:10.1001/jamanetworkopen.2024.8502

eFigure. Absolute Risk for Overall Stroke and Stroke Type by Race and Ethnicity
eTable. Sensitivity Analysis of Association Between Cumulative Mean Systolic Blood Pressure and Time to Incident Stroke in Pooled Cohort Sample by Cohort Subgroup, 1971 to 2019

This supplementary material has been provided by the authors to give readers additional information about their work.

© 2024 Johnson KE et al. *JAMA Network Open*.

eFigure. Absolute Risk for Overall Stroke and Stroke Type by Race and Ethnicity

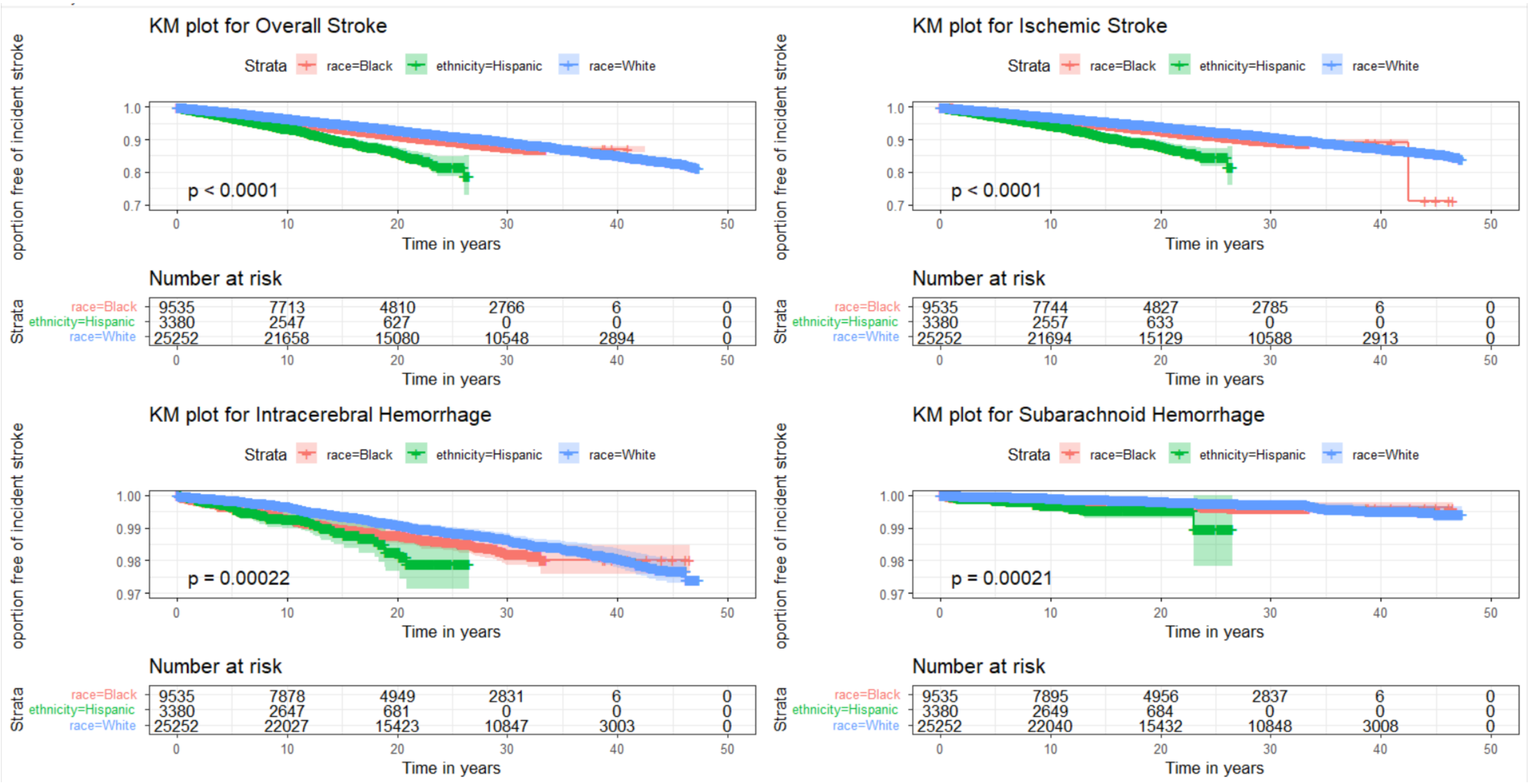

Abbreviations: Kaplan Meier (KM); ICH=Intracerebral Hemorrhage; IS=Ischemic Stroke; SAH=Subarachnoid Hemorrhage;

Stroke risk varied by race and ethnicity. eFigure 1 presents the Kaplan-Meier survival curves for time to incident stroke type by race and ethnicity representing unadjusted hazard ratios. Before covariate adjustment, among the overall stroke and three stroke types, the proportion remaining free of stroke events of Hispanic participants of any race fell more precipitously than for White and Black participants (log rank, *P*<.0001)) (Figure 1a-d). Curves for White and Black participants minimally differed for IS, (Figure 1b), ICH (1c) and SAH (Figure 1d).

© 2024 Johnson KE et al. *JAMA Network Open*.

eTable. Sensitivity Analysis of Association Between Cumulative Mean Systolic Blood Pressure and Time to Incident Stroke in Pooled Cohort Sample by Cohort Subgroup, 1971 to 2019

**a. Multivariable-adjusted Cox Proportional Hazard Regression for the Association between Cumulative Mean Systolic Blood Pressure and Time to Incident Stroke: Combined 6 Cohorts**				
**Types of First Time Incident Stroke**	Overall Stroke<br>HR (95% CI)<br>m=3,502	IS<br>R (95% CI)<br>m=2,952	ICH<br>HR (95% CI)<br>m=448	SAH<br>HR (95% CI)<br>m=98
**Race/Ethnicity**				
Black vs. White	1.22 (1.10, 1.36)<br>P<.001	1.21 (1.08, 1.36)<br>P = .002	1.32 (0.98, 1.78)<br>P = .07	1.61 (0.87, 2.96)<br>P = .13
Hispanic of any race vs. White	1.16 (0.98, 1.39)<br>P=.09	1.08 (0.89, 1.31)<br>P = .42	1.50 (0.90, 2.51)<br>P = .12	3.84 (1.29, 11.40)<br>P = .02
**Race/Ethnicity and Cumulative Mean SBP Interactions** (per 10 mm Hg higher cumulative mean SBP)				
Race/ethnicity^{x}SBP	P= .57	P= .85	P=.02	P= .89
SBP (per 10 mm Hg) among Black	1.22 (1.18, 1.26)<br>P<.001	1.19 (1.15, 1.24)<br>P<.001	1.42 (1.32, 1.53)<br>P<.001	1.09 (0.88, 1.35)<br>P = .45
SBP(per 10 mm Hg) among White	1.20 (1.17, 1.23)<br>P<.001	1.20 (1.17, 1.23)<br>P<.001	1.24 (1.15, 1.33)<br>P<.001	1.15 (0.96, 1.38)<br>P = .12
SBP(per 10 mm Hg) among Hispanic	1.19 (1.14, 1.25)<br>P<.001	1.18 (1.12, 1.24)<br>P<.001	1.30 (1.14, 1.49)<br>P<.001	1.16 (0.91, 1.46)<br>P = .23
**b. Multivariable-adjusted Cox Proportional Hazard Regression for the Association between Cumulative Mean Systolic Blood Pressure and Time to Incident Stroke: ARIC, CARDIA,CHS, and FOS Cohorts Only**				
**Types of First Time Incident Stroke**	Overall Stroke<br>HR (95% CI)<br>m=2763	IS<br>HR (95% CI)<br>m=2321	ICH<br>HR (95% CI)<br>m=364	SAH<br>HR (95% CI)<br>m=77
**Race**				
Black vs. White	1.18 (1.04, 1.34)<br>P = .01	1.18 (1.03, 1.36)<br>P = .02	1.34 (0.96, 1.88)<br>P = .09	1.65 (0.83, 3.28)<br>P = .15
**Race and Cumulative Mean SBP Interactions** (per 10 mm Hg higher cumulative mean SBP)				
Race^{x}SBP	P= .03	P= .47	P= .001	P= .82
Black^{x}SBP	1.27 (1.22, 1.32)<br>P<.001	1.24 (1.19, 1.29)<br>P<.001	1.46 (1.35, 1.58)<br>P<.001	1.14 (0.90, 1.44)<br>P = .27
White^{x}SBP	1.21 (1.18, 1.24)<br>P<.001	1.22 (1.18, 1.25)<br>P<.001	1.22 (1.13, 1.32)<br>P<.001	1.10 (0.90, 1.35)<br>P = .33

© 2024 Johnson KE et al. *JAMA Network Open*.

c. Multivariable-adjusted Cox Proportional Hazard Regression for the Association between Cumulative Mean Systolic Blood Pressure and Time to Incident Stroke: NOMAS, and MESA Cohorts Only				
**Types of First Time Incident Stroke**	Overall Stroke HR (95% CI) m=726	IS HR (95% CI) m=621	ICH HR (95% CI) m=81	SAH HR (95% CI) m=21
**Race/ Ethnicity**				
Black	1.21 (0.98, 1.50) P = .08	1.15 (0.92, 1.45) P = .22	1.07 (0.55, 2.09) P = .84	2.44 (0.40, 14.88) P = .33
White	Ref	Ref	Ref	Ref
Hispanic any race	1.08 (0.85, 1.37) P = .53	0.99 (0.77, 1.28) P = .95	1.18 (0.59, 2.38) P = .64	5.15 (0.86, 30.73) P = .07
**Race/Ethnicity and Cumulative Mean SBP Interactions** (per 10 mm Hg higher cumulative mean SBP)				
Race/ethnicity^{x}SBP	P= .06	P= .06	P= .88	P= .11
Black^{x}SBP	1.10 (1.03, 1.18) P = .003	1.09 (1.01, 1.17) P = .02	1.30 (1.08, 1.55) P = .004	0.93 (0.55, 1.56) P = .77
White^{x}SBP	1.13 (1.05, 1.22) P = .001	1.09 (1.01, 1.19) P = .03	1.38 (1.14, 1.68) P = .001	1.76 (1.13, 2.74) P = .01
Hispanic any race^{x}SBP	1.21 (1.15, 1.27) P<.001	1.19 (1.13, 1.26) P < .001	1.34 (1.17, 1.54) P < .001	1.10 (0.86, 1.42) P = .45

Abbreviations: Atherosclerosis Risk in Communities Study (ARIC), Coronary Artery Risk Development in Young Adults Study (CARDIA), Cardiovascular Health Study (CHS), Framingham Offspring Study (FOS), Multi-Ethnic Study of Atherosclerosis (MESA), and Northern Manhattan Study (NOMAS)

CI=Confidence Interval; HR=Hazard Ratio; ICH=Intracerebral Hemorrhage; IS=Ischemic Stroke; SAH=Subarachnoid Hemorrhage; SBP= Systolic Blood Pressure

m denotes the number of observed events.

In eTable 1a, models for Overall Stroke, IS, and ICH had n= 38167 participants from all the 6 six cohorts with different number of observed events for each stroke type. The model for SAH in eTable 1a had n= 32570 participants from ARIC, CARDIA, FOS, MESA, and NOMAS with observed SAH events m=98.

In eTable 1b, models for Overall Stroke, IS, and ICH had n= 28790 participants from ARIC, CARDIA, CHS, and FOS with different number of observed events for each stroke type. The model for SAH in eTable 1a had n= 23252 participants from ARIC, CARDIA, and FOS with observed SAH events m=77.

© 2024 Johnson KE et al. *JAMA Network Open*.

In eTable 1c, models for Overall Stroke, IS, ICH, and SAH had n= 9318 participants from MESA and NOMAS with different number of observed events for each stroke type.

In eTable 1b, the race/ethnicity*SBP interactions, race/ethnicity modified the association of SBP with overall stroke and time to ICH. The latter result is similar to the final ICH model results for participants in eTable 1a. Therefore, we observed no clear evidence of effect modification of race/ethnicity on first time incident stroke and stroke type. In eTable 1c, Black participants had a higher risk of overall stroke and stroke types than White participants. However, the findings were not significant (*P* >.05).

© 2024 Johnson KE et al. *JAMA Network Open*.
