# Supplementary material for: Cumulative Systolic Blood Pressure and Incident Stroke Type Variation by Race and Ethnicity
Source: JAMA Netw Open. 2024 May 3;7(5):e248502. doi: 10.1001/jamanetworkopen.2024.8502 (PMC11069082; doi:10.1001/jamanetworkopen.2024.8502)
Supplement: Supplement 2. — Data Sharing Statement [file jamanetwopen-e248502-s002.pdf]

## Data Sharing Statement

Johnson. Cumulative Systolic Blood Pressure and Incident Stroke Type Variation by Race and Ethnicity. *JAMA Netw Open*. Published May 03, 2024.
doi:10.1001/jamanetworkopen.2024.8502

**Data**
**Data available:** No
